# Supplementary material for: Predicting proteome dynamics using gene expression data
Source: Sci Rep. 2018 Sep 14;8:13866. doi: 10.1038/s41598-018-31752-4 (PMC6138643; doi:10.1038/s41598-018-31752-4)
Supplement: Supplementary file 1 — Supplementary Information [file 41598_2018_31752_MOESM1_ESM.docx]

**Predicting proteome dynamics using gene expression data**

Krzysztof Kuchta^1,a^, Joanna Towpik^1,a^, Anna Biernacka^1,a^, Jan Kutner^1^, Andrzej Kudlicki^2^, Krzysztof Ginalski^1,*^ and Maga Rowicka^2,*^

^1^Laboratory of Bioinformatics and Systems Biology, Centre of New Technologies, University of Warsaw, 02-089 Warsaw, Poland;

^2^Department of Biochemistry and Molecular Biology, Institute for Translational Sciences, and Sealy Center for Molecular Medicine, University of Texas Medical Branch, Galveston, TX 77555

^a^These authors equally contributed to this work.

^*^To whom correspondence should be addressed. E-mails: kginal@cent.uw.edu.pl, merowick@utmb.edu.

**Appendix. Full derivation of Equation 2.**

We start from Equation 1

$$\frac{d[P_{i}\left( t \right)]}{dt}=k_{trans,i}\cdot\left[ m{RNA}_{i}\left( t \right) \right]-k_{d,i}\left[ P_{i}\left( t \right) \right]$$

and use the substitution

$\left[ R_{i}\left( t \right) \right]=\frac{\left[ P_{i}\left( t \right) \right]}{k_{trans,i}}$,

which yields

$k_{trans,i}\cdot\frac{d[R_{i}\left( t \right)]}{dt}=k_{trans,i}\cdot\left[ m{RNA}_{i}\left( t \right) \right]-k_{trans,i}\cdot k_{d,i}\left[ R_{i}\left( t \right) \right]$.

Dividing both sides by k_trans,I_ gives

$\frac{d[R_{i}\left( t \right)]}{dt}=\left[ m{RNA}_{i}\left( t \right) \right]-k_{d,i}\left[ R_{i}\left( t \right) \right]$.

Integrating both sides from t to $t+\Delta t$ yields

$R_{i}\left( t+\Delta t \right)-R_{i}\left( t \right)=\int_{t}^{t+\Delta t} \left[ m{RNA}_{i}\left( t \right) \right]dt-\int_{t}^{t+\Delta t} k_{d,i}\left[ R_{i}\left( t \right) \right]dt$.

Using the approximation

$\int_{t}^{t+\Delta t} f\left( t \right)dt= \frac{1}{2}\cdot(f\left( t+\Delta t \right)+f(t))\cdot\Delta t$,

we obtain

$R_{i}\left( t+\Delta t \right)-R_{i}\left( t \right)=\frac{1}{2}\cdot\left( \left[ m{RNA}_{i}\left( t+\Delta t \right) \right]+\left[ m{RNA}_{i}\left( t \right) \right] \right)\cdot\Delta t-\frac{k_{d,i}}{2}\cdot\left( \left[ R_{i}\left( t+\Delta t \right) \right]+\left[ R_{i}\left( t \right) \right] \right)\cdot\Delta t$.

Next, we multiply both sides by 2

$${2\cdot R}_{i}\left( t+\Delta t \right)-{2\cdot R}_{i}\left( t \right)=\Delta t\cdot\left( \left[ m{RNA}_{i}\left( t+\Delta t \right) \right]+\left[ m{RNA}_{i}\left( t \right) \right] \right)-k_{d,i}\Delta t\cdot\left( \left[ R_{i}\left( t+\Delta t \right) \right]+\left[ R_{i}\left( t \right) \right] \right)$$

and leave only expressions containing $R_{i}\left( t+\Delta t \right)$ on the left side:

$\left( 2+k_{d,i}\Delta t \right)\cdot R_{i}\left( t+\Delta t \right)=\left( 2-k_{d,i}\Delta t \right)\cdot R_{i}\left( t \right)\Delta t+\Delta t\cdot\left( \left[ m{RNA}_{i}\left( t+\Delta t \right) \right]+\left[ m{RNA}_{i}\left( t \right) \right] \right)$.

Dividing both sides by $\left( 2+k_{d,i}\Delta t \right)$we obtain the final form of Equation 2:

$$\left[ R_{i}\left( t+\Delta t \right) \right]=\frac{2-k_{d,i}\cdot\Delta t}{2+k_{d,i}\cdot\Delta t}\cdot\left[ R_{i}\left( t \right) \right]+\frac{\Delta t}{2+k_{d,i}\cdot\Delta t}\cdot\left( \left[ m{RNA}_{i}\left( t+\Delta t \right) \right]+\left[ m{RNA}_{i}\left( t \right) \right] \right).$$
